# Parametric studies of metabolic cooperativity in *Escherichia coli* colonies: Strain and geometric confinement effects

**DOI:** 10.1371/journal.pone.0182570

**Published:** 2017-08-18

**Authors:** Joseph R. Peterson, John A. Cole, Zaida Luthey-Schulten

**Affiliations:** 1 Department of Chemistry, University of Illinois at Urbana-Champaign, Urbana, IL, United States of America; 2 Department of Physics, University of Illinois at Urbana-Champaign, Urbana, IL, United States of America; 3 Carl R. Woese Institute for Genomic Biology, University of Illinois at Urbana-Champaign, Urbana, IL, United States of America; 4 Beckman Institute for Advanced Science and Technology, University of Illinois at Urbana-Champaign, Urbana, IL, United States of America; University of Helsinki, FINLAND

## Abstract

Characterizing the complex spatial and temporal interactions among cells in a biological system (*i.e*. bacterial colony, microbiome, tissue, *etc*.) remains a challenge. Metabolic cooperativity in these systems can arise due to the subtle interplay between microenvironmental conditions and the cells’ regulatory machinery, often involving cascades of intra- and extracellular signalling molecules. In the simplest of cases, as demonstrated in a recent study of the model organism *Escherichia coli*, metabolic cross-feeding can arise in monoclonal colonies of bacteria driven merely by spatial heterogeneity in the availability of growth substrates; namely, acetate, glucose and oxygen. Another recent study demonstrated that even closely related *E. coli* strains evolved different glucose utilization and acetate production capabilities, hinting at the possibility of subtle differences in metabolic cooperativity and the resulting growth behavior of these organisms. Taking a first step towards understanding the complex spatio-temporal interactions within microbial populations, we performed a parametric study of *E. coli* growth on an agar substrate and probed the dependence of colony behavior on: 1) strain-specific metabolic characteristics, and 2) the geometry of the underlying substrate. To do so, we employed a recently developed multiscale technique named 3D dynamic flux balance analysis which couples reaction-diffusion simulations with iterative steady-state metabolic modeling. Key measures examined include colony growth rate and shape (height vs. width), metabolite production/consumption and concentration profiles, and the emergence of metabolic cooperativity and the fractions of cell phenotypes. Five closely related strains of *E. coli*, which exhibit large variation in glucose consumption and organic acid production potential, were studied. The onset of metabolic cooperativity was found to vary substantially between these five strains by up to 10 hours and the relative fraction of acetate utilizing cells within the colonies varied by a factor of two. Additionally, growth with six different geometries designed to mimic those that might be found in a laboratory, a microfluidic device, and inside a living organism were considered. Geometries were found to have complex, often nonlinear effects on colony growth and cross-feeding with “hard” features resulting in larger effect than “soft” features. These results demonstrate that strain-specific features and spatial constraints imposed by the growth substrate can have significant effects even for microbial populations as simple as isogenic *E. coli* colonies.

## Introduction

Metabolic competition and cooperativity are ubiquitous in nature with recent research reaffirming the old adage: location is everything. Whether it be the division of labor among the cells in an animal body or the complex chemical warfare among a soup of bacteria competing for limited resources, spatial and temporal variation are key factors which must be understood. Interactions in microbial communities, which often comprise tens to hundreds of metabolically distinct species [1], are of particular interest in areas ranging from human health [2] to ecology of the world’s nutrient cycles [3, 4]. These communities form complex networks of cooperative and competitive interactions that ultimately determine the population’s dynamics, steady-states, and robustness to change [5]. For example, it is now known that among people with chronic bowel diseases, the composition of the gut microbiota can vary considerably relative to the “healthy” patient [6, 7]. More generally, the potential for emergent metabolic cooperativity via a wide array of organic acids, inorganic molecules, salts, and purine/pyrimidines have been predicted in two-member bacterial communities [8]. Underlying the stability and structure of these populations are a complex network of metabolic and physical interactions that vary both spatially and temporally [9]; thus, part of what is needed to understand how these populations behave is an understanding of the metabolism of community members growing alone and in concert with their neighbors.

A number of computational systems biology techniques have been developed for understanding the growth and metabolic requirements of different microbes. Chief among these is a family of methods based on flux balance analysis (FBA) [10]. FBA describes the steady-state growth and fluxes within an organism’s metabolic network subject to internal constraints (*e.g*. reaction upper bounds proceeding from the finite copy numbers of catalytic enzymes and their turnover rates) and external constraints (*e.g*. limited availability of a molecule needed for growth). Several FBA methods designed to examine communities of microorganisms have been developed including community FBA (cFBA), OptCom, dynamic multi-scale FBA (dmsFBA) and population FBA [8, 11–14]. cFBA compartmentalizes different organisms but allows them to compete for and exchange metabolites through an extracellular compartment [12]. OptCom uses a similar technique, however it allows for a community objective to be defined, which enables users to identify optimal engineering strategies for a community [11]. dmsFBA relaxes the common steady-state assumption in order to simulate how cross-feeding evolves with time [8]. Population FBA simulates metabolic phenotypes in populations of microbes that arise due to capacity constraints that arise from stochastic gene expression [13]. For an excellent review of these and other community methods and studies, see [15]. All of these methods are limited in that they treat the entire population as being “well-stirred”, that is, seeing the same chemical and spatial environment. This may be a reasonable approximation for some problems (e.g. bioreactors), but spatial heterogeneity is innate to many important scenarios (*e.g*. biofilms, microbiome).

**Fig 1:**
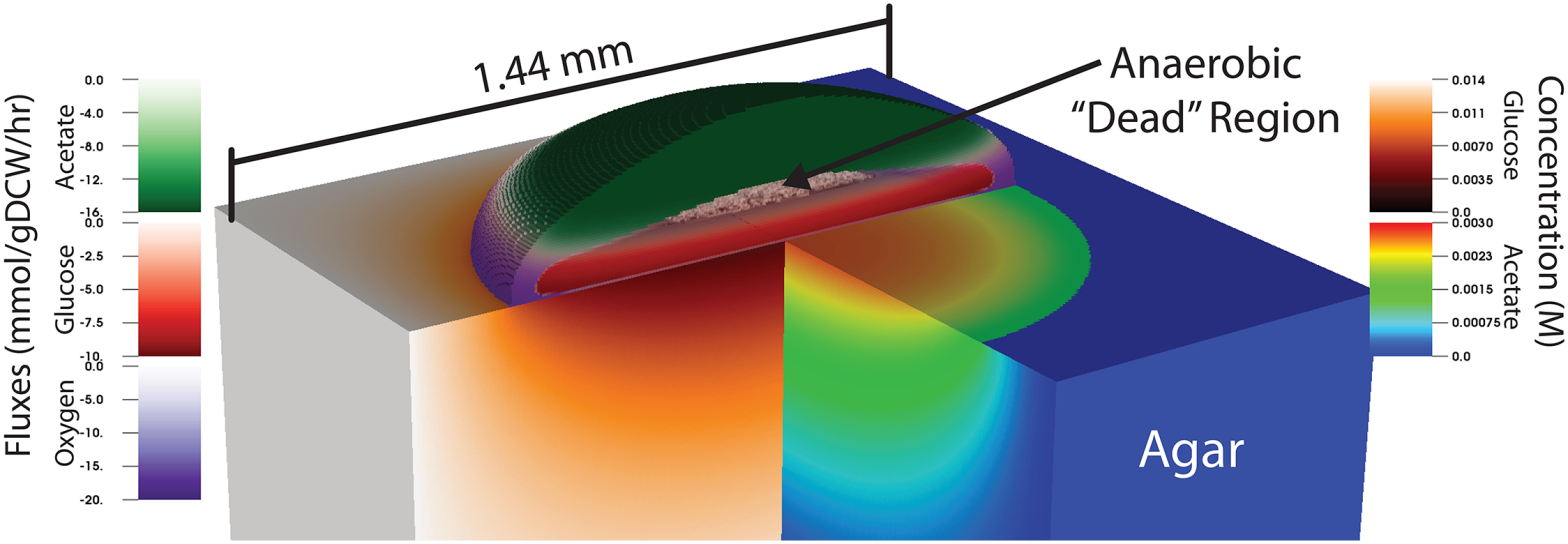
Metabolic crossfeeding in *E. coli*. A visualization of a 3DdFBA simulation of metabolic cooperativity in *E. coli* K-12 MG1655 growing on a 2.5% glucose agar substrate (described previously [16]). The image depicts metabolic cooperativity between aerobic (purple) and anaerobic (red) glucose utilizing cells, which generate acetate that is utilized aerobically by glucose starved cells (green). Complete substrate utilization at the top and bottom of the colony create an anoxic dead zone containing essentially dormant cells. Color bars on the left represent metabolic fluxes of the indicated metabolites while those on the right indicate concentrations of glucose and acetate inside the agar. The image was visualized using the VisIt visualization software (as described in the Methods) with a custom plugin written to support 3DdFBA simulation.

A recently developed multi-scale systems biology technique provides a means to examine the spatial dependence of microbial growth. Named 3-dimensional dynamic flux balance analysis (3DdFBA) [16], this method solves a reaction-diffusion equation on a lattice, allowing chemicals to diffuse in and among various phases (*i.e*. solid, liquid, gas, cell, *etc*.) and be taken up by cells living in a given lattice site, effectively converting chemicals into biomass. The method has been formulated with both stochastic [17, 18] and continuous [16] descriptions of the reaction and diffusion of the chemical species. The stochastic version of the method was applied to small colonies (∼100 cells) of *E. coli* growing in a micro-aerobic environment [17, 18]. These simulations suggested that metabolic cooperativity could arise in an isogenic *E. coli* population, which in turn prompted the development of a continuous version capable of simulating macroscopic colonies [16]. Laboratory scale simulations of *E. coli* colonies growing on 2.5% glucose minimal media predicted that after sufficient time the population would fractionate into glucose-utilizing cells that ferment acetate, and acetate-utilizing cells which are starved of glucose (Fig 1). A number of other spatially-resolved FBA methods have also been developed, and used to study mutualism and competition in two dimensional multispecies communities (COMETS; [19]) as well as chronic wound biofilm homoeostasis (DFBALab; [20, 21]).

These spatially-resolved methods have the potential to add new relevance to computational biology by bridging temporal and spatial scales that are currently difficult or impossible to study experimentally. As a demonstration of their utility for complex 3D geometries, we performed parametric studies of metabolic cross-feeding using 3DdFBA in: 1) colonies of closely related *E. coli* strains, and 2) colonies grown on agars with geometries other than merely flat surfaces. We also analyze the error resulting from the discretization of the reaction-diffusion equation and show that grids finer than 20 μm are required to ensure converged solutions. Our simulations demonstrate that 3DdFBA can predict significant effects on the dynamics of microbial populations that arise through subtle differences between strains. Finally, we show that while abrupt changes in the shape of a colony’s substrate (*i.e*. the existence of a nearby wall of agar) can dramatically impact the colony’s growth, more subtle curvatures (such as those that may arise within the gut) give rise to growth dynamics that are very similar to reference colonies grown on flat agar.

## Materials and methods

### 3DdFBA

Three dimensional dynamic flux balance analysis (3DdFBA) is only briefly reviewed here, as it was described rigorously previously [16]. The method couples a partial differential equation (PDE) description of chemical transport with flux balance analysis (FBA) [10]. Broadly speaking, the PDE represents the chemical species while FBA represents the cells. Metabolites are consumed and produced by a reaction-diffusion PDE:

$$\begin{array}{c} \frac{\partial \vec{C}}{\partial t}=D\nabla^{2}\vec{C}+R\left(\vec{C}\right) \end{array}$$ (1)

where $\vec{C}$ is a vector containing the concentrations of metabolites, **D** encodes the diffusion rates of the metabolites and $R(\vec{C})$ encode the reactive fluxes of the species. *R* includes any reactions among chemical species, active and passive transport into and out of cell volume and, crucially, exchange fluxes computed via a local dynamic FBA (dFBA) [22] simulation (more precisely, fluxes are read from a table of solutions computed via FBA and the solution is used to compute uptake and efflux). Within this study, the reaction diffusion equation is solved on a 3 dimensional regular cubic lattice via a central finite difference scheme [23]. Dirichlet boundary conditions (constant value) are applied for all chemical species on the boundaries of the simulation volume. Gaseous species are allowed to diffuse anywhere in the simulation domain, while aqueous molecules (acetate, glucose, etc.) are limited to diffusion in lattice sites containing agar and cell mass.

Cells, while being represented as a volume fraction (*ϕ*_*i*_) on the lattice, are not diffused or actively transported (e.g. via chemotaxis) among lattice points. Rather, as cell growth occurs they are pushed isotropically into neighbouring lattice points (after some maximum volume fraction within the lattice site is achieved, namely ∑_*i*_ *ϕ*_*i*_ ≥ 0.65). Volume fraction is related the mass of cells as

$$\begin{array}{c} \phi_{i}=\frac{m_{i}}{V\rho_{i}} \end{array}$$ (2)

where *m*_*i*_ is the mass of a particular cell type in the lattice site, *V* is the volume of the lattice site, and *ρ*_*i*_ = *m*_*i*,*cell*_/*V*_*cell*_ is the density of a single cell with *m*_*i*,*cell*_ and *V*_*cell*_ taken to be 258 fg and 1 fL [24], respectively. Cell mass grows exponentially at the rate set by the local dFBA as

$$\begin{array}{c} \frac{dm_{i}}{dt}=v_{bm,i}m_{i} \end{array}$$ (3)

where *v*_*bm*_ is the flux through the biomass equation. An absorbing boundary condition for the cell mass is applied to the boundaries of the simulation volume. Cell mass is prevented from penetrating into the agar substrate. The reaction term in Eq 1 is coupled to the cell mass and the predicted uptake flux as $R\left(\vec{C}\right)=\vec{m}\cdot {\vec{v}}_{C}$ where ${\vec{v}}_{C}<{\vec{v}}_{C,max}$. The maximal uptake/secretion rate, *v*_*C*, *max*_, is constrained assuming enzyme saturation effects (e.g. Michaelis-Menten kinetics for glucose uptake) and to prevent a chemical in a lattice site from becoming negative ($\vec{C}\geq 0$). The cell volume fraction couples to the chemical concentrations by hindering diffusion (*e.g*. an attenuated diffusion rate computed according to a diffusion law that considers the local cell volume fraction [25]) and via the reaction term discussed above. Volume fractions (*ϕ*_*i*_) for each cell phenotype are tracked at each lattice site, and a “regulation” function allow cells to transition between phenotypic states depending on the local concentrations of the chemical species. As an example, glucose utilizing cells can be converted into acetate utilizing cells if there is plentiful acetate but no glucose available at the lattice site for a significant amount of time. See [16] for more description of the “regulation” function.

Analysis, simulation and visualization codes to simplify the analysis of 3DdFBA simulation were created for this study. These codes can be found at http://www.scs.illinois.edu/schulten/. An analysis of the performance and memory requirements of the code can be found in S1 Fig. Visualization in Fig 1 was performed using a custom plugin to the VisIt visualization software [26].

### *E. coli* strains

We selected five *E. coli* strains with curated genome scale metabolic models (GEMs) [27–29] for characterization: BL21, Crooks, MG1655, W and W3110. Chemostat experiments demonstrated variable glucose utilization efficiency and acetate production rates among these strains [29]. Additionally, some strains produce acetate during aerobic glucose growth, while others do not. Models for the *E. coli* strains (*i*JO1366, *i*B21_1397, *i*EcolC_1368, *i*WFL_1372 and *i*Y75_1357) were obtained from the BiGG Models database version 1.3 [30]. All FBA simulations were performed using COBRApy [31]. For all simulations, the core *E. coli* biomass reaction [32] was used as the primary objective reaction.

When used unmodified, the models were incapable of predicting the correct growth rate and acetate production rate (neither aerobically nor anaerobically) when setting glucose uptake rates to those measured in the chemostat experiments. Therefore, the models were adjusted to minimize errors in acetate production and growth rates under both aerobic and anaerobic conditions. This was accomplished by fitting the maximum oxygen uptake rate and growth associated maintenance (ATP cost for cell growth) to experimental data. The fit parameters, glucose uptake rate, predicted acetate and growth rates, and the associated errors are shown in Table 1. In general, growth and aerobic acetate production rates could be fit with little error. The anaerobic acetate production rate was more difficult to capture; over the five strains we found an average error of 16.1%.

**Table 1:** Growth characteristics of models of the *E. coli* strains examined in the current study. Results are shown for models [27–29] where maximal O_2_ and growth associated ATP maintenance have been fitted to minimize deviation from experimental acetate production and growth rates.

Strain	Growth Rate^a^		*v*_*glucose*_^b^		*v*_*acetate*_^c^		*v*_*o*2_^d^	GAM^e^	
	+O_2_^f^	−O_2_^g^	+O_2_	−O_2_	+O_2_	−O_2_		+O_2_	−O_2_
**B21**	0.76 (0%)^h^	0.29 (0%)	-8.0±0.3	-11.3±0.5	0.0 (0%)	9.17 (3.9%)	-15.5	60.25	50.45
**Crooks**	0.96 (0%)	0.77 (0%)	-12.5±0.5	-30.9±2.2	0.0 (0%)	25.2 (32%)	-33.5	121.45	59.75
**MG1655**	0.84 (15.2%)	0.46 (0%)	-9.5±0.3	-16.7±0.2	3.49 (0%)	13.27 (13.3%)	-13.9	60.25	50.45
**W**	0.97 (0%)	0.9 (0%)	-9.9±0.1	-27.2±1.4	0.0 (0%)	20.53 (2.6%)	-17.8	54.55	35.65
**W3110**	0.61 (0%)	0.52 (0%)	-6.7±0.1	-17.5±0.5	3.03 (2.7%)	13.63 (28.7%)	-7.5	36.65	41.65

^a^Optimal growth rate for cells grown in a chemostat in units of hr^-1^.

^b^Experimentally quantified maximal uptake rate for glucose in units of mmol/gDCW/hr [29].

^c^Maximal efflux rate for acetate in units of mmol/gDCW/hr.

^d^Fitted maximal uptake rate for oxygen in units of mmol/gDCW/hr.

^e^Fitted growth associated maintenance cost in units of mmolATP/gDCW.

^f^+*O*_2_—Aerobic growth.

^g^−*O*_2_—Anaerobic growth.

^h^Values in parenthesis indicate percentage error compared to experimentally measured values after fitting.

Two phenotypes of *E. coli* were considered in the simulations: 1) those growing aerobically on glucose, and 2) those growing aerobically on acetate. Tables of FBA solutions (including uptake and efflux of key metabolites and growth rates) were generated for the phenotypes for all five *E. coli* strains. Because strain-specific acetate consumption data was not available, the maximal acetate uptake rate used in [16] was adopted. FBA tables were generate with 50 (160) divisions between 0 and the maximal glucose (oxygen) uptake rates.

### Spatial geometries

**Fig 2:**
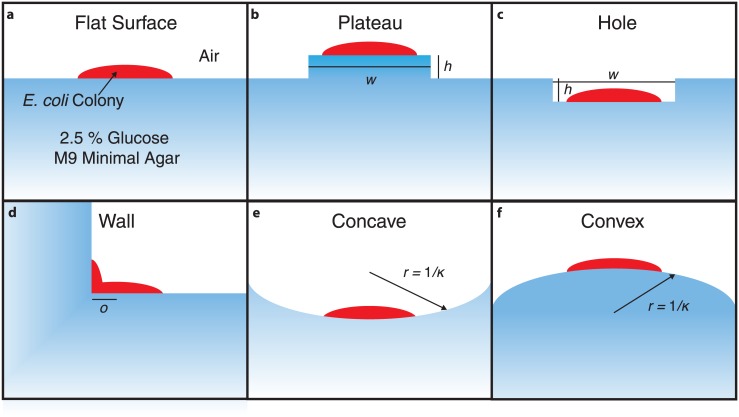
Geometries investigated. An illustrative schematic showing the six geometries examined in this study. Various characteristic variables of the geometry are indicated with symbols. See the Methods and Results text for numerical values and descriptions of these variables.

Spatial geometries that mimic engineered and natural growth environments were selected for analysis (Fig 2). A flat surface intended to mimic the standard agar in a Petri dish has been studied previously [16]. Additional geometries, namely the “plateau”, “hole” and “wall”, were designed to represent geometries that might be encountered in a microfluidic device (for instance in a microwell). For the plateau and hole geometries, two parameters were varied, namely the height (*h*) and width (*w*). For simplicity, the plateau and hole were taken to be square. For the wall geometry, the offset of the initial colony seed from the colony edge (*o*) is the only parameter. This parameter is of interest as the onset of metabolic cooperativity occurs after the colony has grown to some initial size and thus allows the investigation the effect of a confinement on onset. The final two geometries we studied include concave and convex surfaces that are designed to mimic biological systems such as the inside of an intestine, or the surface of a rough skin. For the sake of simplicity, these geometries had uniform curvature defined through a radius parameter.

## Results & discussion

### Resolution dependence of 3DdFBA solution

The ability to resolve features within a 3DdFBA simulation depends on the resolution of the grid used to represent chemical concentrations and cell fractions. This resolution dependence is non-trivially dependent on diffusion and reaction rates, and on physical boundary conditions (*e.g*. the agar surface in the simulation). We examined how several features of interest (*e.g*. fluxes, chemical concentrations, and cellular phenotypes) differ across a range of grid resolutions.

As a test system, we simulated the previously published *E. coli* K-12 MG1655 model [16] at eight grid resolutions ranging from 4.1 to 120 μm. Each colony simulation was seeded with the equivalent of a single cell’s mass on the surface of the agar in the center of the simulation domain. Growth of the resulting colony was simulated for 40 hours to allow sufficient metabolic cooperativity to arise. Colonies grew to fill the agar and began to interact with the simulation boundary conditions at about 35 hours of growth. After this simulations began to exhibit boundary effects due to the use of fixed-concentration (Dirichlet) boundary conditions. Therefore, the resolution dependence was examined at the 30 hour time-point before any significant boundary effects arose.

**Fig 3:**
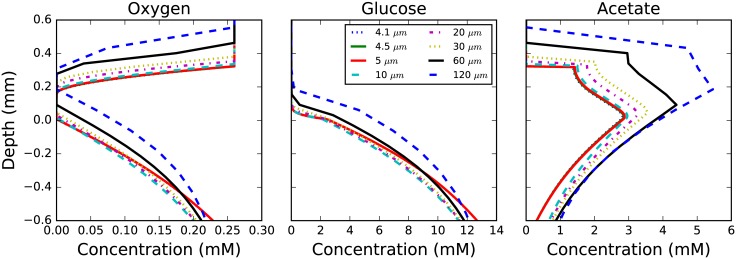
Species profiles. Profiles through the center axis of an *E. coli* K-12 MG1655 colony showing the concentrations (x-axis) of several metabolites at a given depth (y-axis) relative to the agar surface after 30 hours of colony growth. Simulations demonstrate that concentrations predicted by 3DdFBA have a strong dependence on the grid resolution. Concentration profiles are essentially converged after 10 μm; the deviation below a depth of about −0.2 mm seen for higher resolutions (4.1 to 5 μm) is due to boundary condition effects as a thinner agar layer was needed to allow for the simulation to fit in GPU memory.

**Fig 4:**
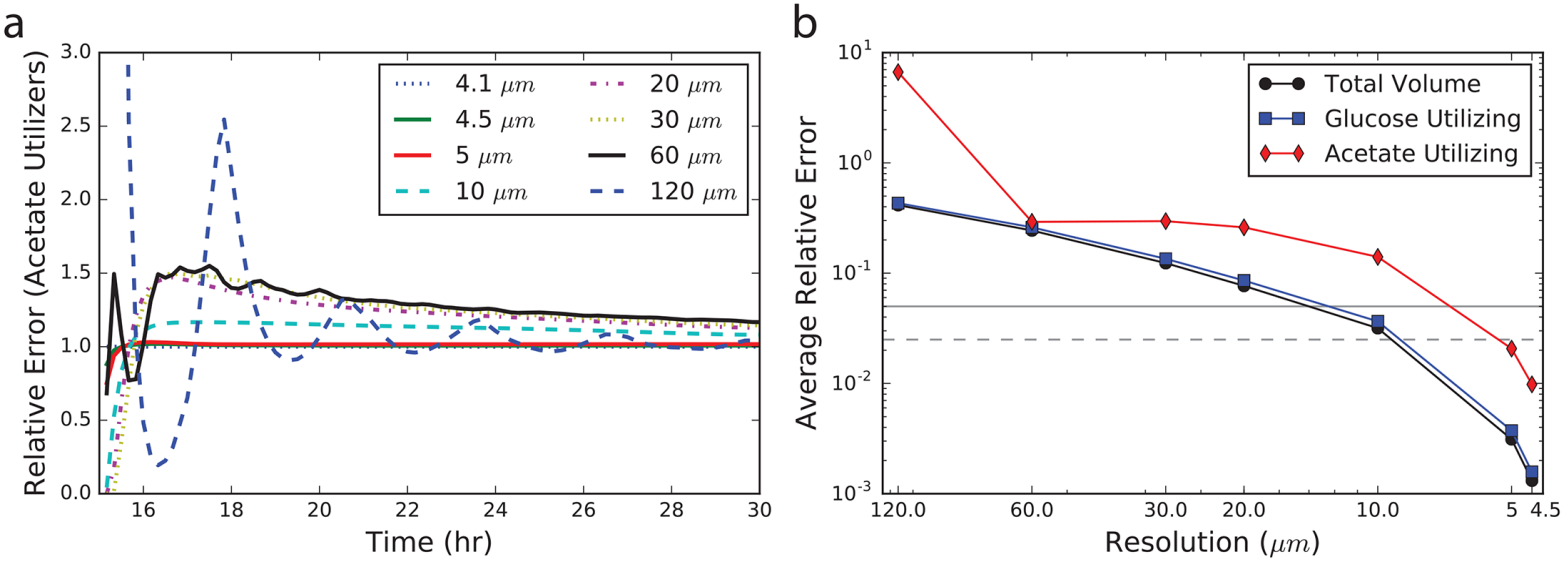
Error analyses. a) Error in the acetate utilizing population fraction, relative to the 4.1 μm simulation, after the emergence of metabolic cooperativity (occurring at about 15 hours). Coarse resolutions introduce numerical oscillations when coarser than about 20 μm. b) Error in the computed volume of cells in an *E. coli* K-12 MG1655 colony as a function of grid resolution. Errors are computed as the relative deviation from a simulation with a 4.1 μm grid resolution averaged from 10 to 30 hours of colony growth. Total colony volume (black circles), volume of glucose utilizing cells (blue squares) and volume of acetate utilizing cells (red diamonds) are shown. Horizontal lines show 5% (solid) and 2.5% (dashed) error in the volumes.

We examine first the resolution dependence of the chemical concentration profiles within the colony; this particular feature is important in driving the partitioning of community members into different metabolic phenotypes during the simulation (Fig 3). Profiles taken through the colony’s central axis show that the concentration profiles sharpen and the colony height narrows as finer grid resolutions are employed (compare profiles between −0.2 and 0.4 depth). This spreading as the resolution is coarsened allows the acetate utilizing fraction of the colony to grow more quickly and to a larger overall fraction of the total colony composition (see Fig 4a). The acetate concentration profile is especially illustrative of the resolution dependence (see Fig 3 (right), showing how the structure changes as the resolution is coarsened. Encouragingly, the profiles do converge as the resolution is increased.

Perhaps more pertinent to questions regarding metabolic cross-feeding is the population composition; therefore, we next examined the error in the predicted fractions of glucose- and acetate-utilizing cells as a function of the grid resolution (Fig 4b). As no analytical solution to the problem is known, we compute the error relative to the finest grid resolution simulation (*i.e*. 4.1 μm). The average relative error was computed over the period from 10 to 30 hours of growth as this is when the initial expansion and differentiation occurs in the colony. The results demonstrate that the error rapidly converges, and that below approximately 10 μm grid spacing, the errors in the population fractions fall below <10% (Fig 4b). Of special note is the fact that when resolutions become large, error in the population fractions rapidly increases and in fact some numerical instability—as demonstrated by the non-physical oscillatory solution—emerges with grid resolutions coarser than 20 μm (Fig 4a). Previous spatially-resolved FBA studies have examined mixed-species simulations with grid resolutions ranging from 200μm to ∼500μm [19]. Our results show that in order to simulate processes in which the reaction to diffusion ratios of metabolites are similar to those of glucose and acetate, finer grid resolutions must be used. While we did not study the resolution dependence of any of the previous studies, our results suggest that at least some of them may not have been simulated at a resolution adequate to ensure reasonably converged results. Nevertheless, we note that our results are for a colony growing at the boundary of two different phases (*i.e*. agar/cells and air), one of which occludes certain metabolites, and that the convergence characteristics might therefore be different from previous studies.

### *E. coli* strain-dependent features of acetate cross-feeding

Different *E. coli* strains have evolved different glucose utilization rates (see Table 1), presumably due to some environmental (or engineered, in the case of commercial strains) stress. While aerobic and anaerobic growth rates were highly correlated with glucose utilization rates (p<0.05 and p<0.02, respectively), the aerobic and anaerobic growth rates were not significantly correlated (p>0.19). Guessing what cross-feeding behavior will arise due to these differences is nontrivial. To complicate the issue, two of the strains produce acetate via overflow metabolism when grown aerobically, the rates of which are uncorrelated with the acetate production rate when grown anaerobically.

We hypothesized these differences could give rise to strain-specific cross-feeding features which 3DdFBA simulations could predict. Models for each of the organisms were obtained from the BiGG database [30] and fit to measured aerobic and anaerobic growth and acetate secretion rates [29]. The fit models exhibited very low error in the growth and aerobic acetate secretion rates and moderate error in anaerobic acetate production rates (see Table 1 and Methods for a detailed analysis of the models). Each strain was simulated on a flat agar surface with identical initial conditions for 50 hours of growth with a grid resolution of 10 μm to ensure a converged answer.

Overall, colonies grew at different rates due primarily to their differences in substrate utilization rates. Generally, glucose diffusing up through the agar was depleted by the cells growing at the periphery and bottom of the colony, while oxygen diffusing in from above was predominantly consumed by those at the top of the colony (producing an anoxic zone with little growth at the colony center). Heatmaps depicting concentrations and fluxes of the major metabolites in a slice through the colony center can be seen in S2 Fig The structure of the actively growing cells (depicted by high metabolic flux in the S2 Fig) are generally the same, though several significant differences can be seen (for instance, compare oxygen and glucose uptake rates for strains W3110 and BL21). Counter-intuitively, height to width ratios of the colonies varied by at most 22% early on, and settled down to a maximum difference of 12% (see S3 Fig).

**Fig 5:**
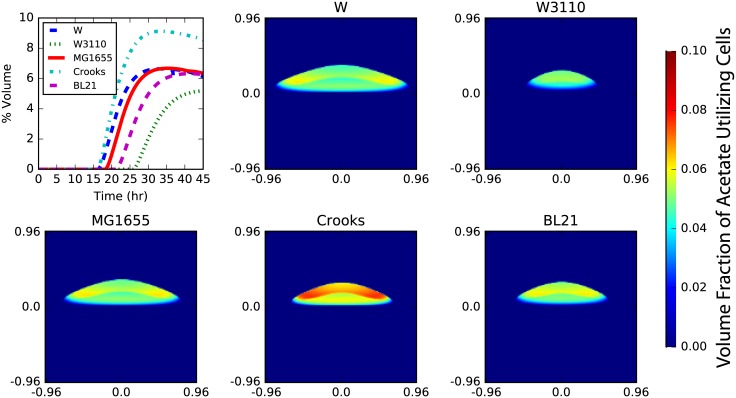
Strain dependence of crossfeeding. Differences in metabolite utilization efficiency of closely related *E. coli* strains give rise to differences in metabolic cross-feeding. Cross-sections of the acetate utilizing volume fraction of cells after 30 hours of growth show that, while the colony growth rates are slightly different, the structure of the metabolic cross-feeding is essentially the same (heatmaps). A quantification of colony volume, however, shows that the fraction of colony that utilize acetate can vary by a factor of 2 and the timing for onset of metabolic cooperativity can vary by up to 10 hours (top left). The acetate utilizing fraction are cells that have transitioned into the phenotypic state where they can catabolize acetate; while these cells are not necessarily consuming acetate, they have the capacity to do so. In general, about 70% of these cells are consuming acetate and are doing so at the maximum uptake rate.

Acetate cross-feeding naturally arose in simulations of all *E. coli* strains (Fig 5). While the structural profiles of the colony (*i.e*. the spatial arrangement of acetate to glucose utilizing populations) were similar among the strains, several features did vary significantly. Specifically, the timing of the onset of the cross-feeding and the partitioning of the colony members into metabolic phenotypes differed significantly (see Fig 5; top left). The timing of the emergence of an acetate utilizing fraction varied by ∼10 hours, ranging from 15 to 25 hours after inoculation. This time appears to primarily be set by the rate of aerobic growth on glucose (*cf*. S1 Table, Fig 5 and S3 Fig). This can be understood simply to be a matter of how much time is required for the colony to grow tall enough such that the glucose entering the bottom is metabolised before it reaches the top. Partitioning among the metabolic cooperators also shows a high degree of strain dependence. After the onset of metabolic cooperativity, the acetate utilizing fraction of the population quickly rises to some (nearly) steady-state. Acetate utiliziers were found to comprise between 5 and 10% of the colony by volume (of which ∼70% are actually consuming acetate). The Crooks *E. coli* strain had the largest fraction of acetate utilizers while the W3110 strain had the smallest fraction. The other three strains had similar acetate utilizing fractions of about 6 and 7%. Quantitatively, the strains shown in Fig 5 have significantly different chemical turnover; for instance Crooks (W3110) has ∼40% higher (lower) acetate turnover after accounting for differences in colony volume (see S4 Fig).

To identify the sources of differences between strains, correlations were computed between various strain-dependent characteristics and both the maximum acetate utilizing fraction and the onset time (see S1 Table). We found that the onset time and overall acetate fraction were set by qualitatively different strain features. Specifically, the onset time was primarily controlled by the maximal aerobic growth rate (with faster-growing strains having earlier onset times), while the overall acetate utilizing fraction was only weakly correlated with the aerobic and anaerobic growth rates. The acetate utilizing fraction turned out to be highly anti-correlated with the maximal acetate uptake rate. In essence, the onset time depends on how quickly the colony grows large enough to have an anoxic region in the interior, while the thickness of the acetate utilizing fraction depends on how much of the colony is acetate-rich, which in turn depends on how fast cells deplete the available acetate. We found that gene expression values for malate dehydrogenase (mdh), succinyl-CoA synthettase (sucCD), and 2-oxoglutarate dehydrogenase (sucAB) were correlated with anaerobic flux values through their reactions (a similar result was identified in [29]), and could be the sources of differences in growth rates and acetate production rates in the different strains. More practically, we found that the overall acetate production rate (*i.e*. loss of acetate to the environment) was highly correlated with the acetate utilizing fraction.

Subtle differences in strain growth, when considered in the context of microbial communities, could give rise to drastically different population dynamics. For instance, competition for acetate with another microbe could be drastically affected by the onset time for metabolic cross-feeding. Additionally, partitioning of the colony into different phenotypes could effect its robustness to environmental changes or stresses. While we did not study these effects here, it is clear that the 3DdFBA methodology could be used for their investigation.

### Geometry dependence of acetate cross-feeding

**Fig 6:**
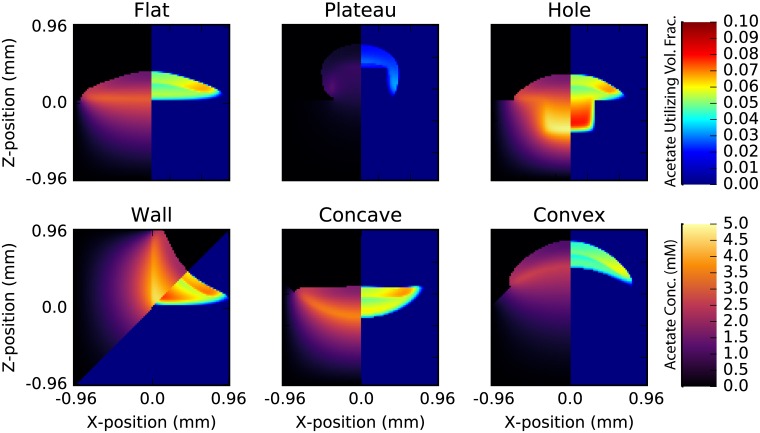
Geometry dependence of crossfeeding. Snapshots of each of colonies after 35 hours of growth in six different geometries showing the drastic difference in growth rate and acetate cross-feeding caused by agar geometry. Each image shows acetate concentration (left) and volume fraction of acetate utilizing cells (right). The acetate utilizing fraction are cells that have transitioned into the phenotypic state where they can catabolize acetate; while these cellsaare not necessarily consumping acetate, they have the capacity to do so. In general, about between 45 and 76% of these cells are consuming acetate and are doing so at the maximum uptake rate.

The cross-feeding behavior in *E. coli* depends on characteristics intrinsic to the particular strain (*i.e*. growth rate, maximum uptake and efflux rates, metabolic efficiency, *etc*.) as well as interactions with the environment (*i.e*. structural confinement and availability of nutrients). To examine the latter effect, we simulated *E. coli* K-12 MG1655 growing on agar surfaces with various geometric features (see Fig 2). A grid resolution of 10 μm were used for all simulations, and the colony was seeded in the center of the computational domain. Characteristic images of simulations of these geometries can be seen in Fig 6. Significant differences in colony growth rate and acetate cross-feeding are apparent in the figure. Specific features of each geometry will be described in turn.

#### Wall

**Fig 7:**
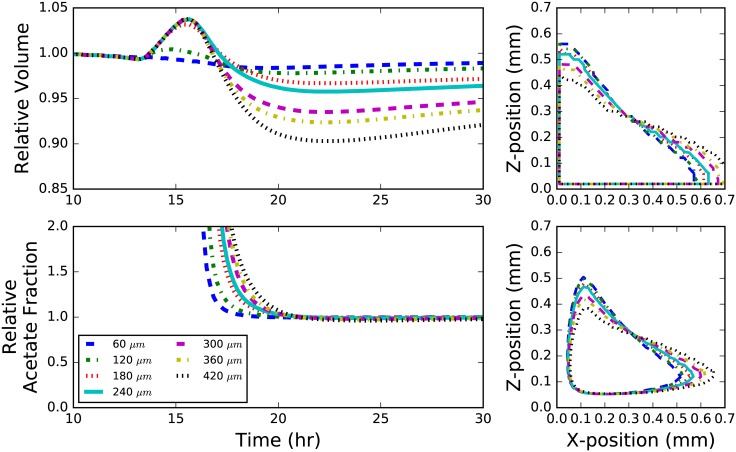
Wall geometry. Behavior of *E. coli* colonies grown next to an agar wall containing metabolizable glucose. Different curves indicate the distance of the initial colony seed from the wall. Colonies grown further from the wall generally grow slower than colonies seeded near the wall, however tend to grow more quickly when they interact with the wall as demonstrated by an inflection in each curve (top left). The fraction of acetate utilizers in a community linearly on the distance from the wall (bottom left). A profile the colony showing the acetate utilizing fraction (contour level = 0.05; bottom right) and the whole colony (contour level = 0.64; top right) after 25 hours of growth. Colony volume and acetate fraction are shown relative to a colony that began growth at the edge where the wall meets the floor.

Colonies were grown on an agar surface with starting distances ranging between 0 to 210 μm from a 90 degree agar wall (see Fig 7). In this geometry, the colony grows until it interacts with the agar wall, which not only provides a physical barrier, but also an additional reservoir of glucose to adjacent cells. As a result, colonies seeded closer to the wall tended to grow more quickly in general (and asymmetrically in the direction of the wall), and the rate of colony growth increased after interaction with the wall (up to 10% greater; see Fig 7; top left). The onset of acetate cross-feeding varied only by about 3 hours; however, the fraction of total cells in a colony that utilized acetate was relatively unchanged after a about 20 hours of growth (Fig 7; bottom left).

Two especially interesting features are seen in the wall geometry simulations. First, while the agar surface area is the same, the colonies tend to grow more quickly than they otherwise would a flat surface. Second, an additional acetate utilizing fraction forms near the wall edge starting after about 20 hours (see Fig 6). This acetate fraction grows significantly faster than the one seen in flat surface colonies, and leads to a larger overall acetate volume fraction by about 25%. The formation of this second fraction is due primarily to the large build-up of acetate in the center of the colony. A similar effect is seen in the hole geometry discussed later.

#### Hole and plateau

**Fig 8:**
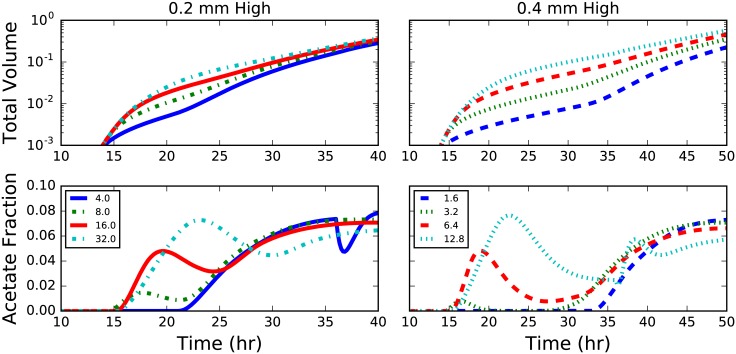
Plateau geometry. Plots of total colony volume (top) and fraction of acetate utilizers (bottom) for colonies grown on plateaus of agar 0.2 (left) and 0.4 (right) mm in height. The legend indicates the aspect ratio (width:height) of the plateau and colors correspond to simulations with the same plateau width.

Colonies growing on the top of plateaus and at the bottom of holes exhibit the most complex dynamics of any in this study. For plateaus, the height is the most important role, primarily because glucose becomes severely limited as the column grows taller (*cf*. left vs. right in Fig 8). Growth significantly slows after the glucose in the column is consumed (Fig 8; top right). Shorter columns lead to a significant decreases in the acetate utilizing fractions after the colonies have grown down the sides of the plateau and begin to interact the substrate below. After some time this effect begins to ebb, and the acetate fraction increases again, settling to a steady state near 8% (slightly larger than that of a colony grown on a flat surface). The duration and extent of this transient drop in acetate utilixing fraction depends strongly on the aspect ratio of the plateau; wider plateaus exhibit more moderate transients (less of a dip, see Fig 8; bottom). Wider plateaus also exhibit faster growth rates (Fig 8 top), primarily due to the longer expansion time before having to grow around the edge.

**Fig 9:**
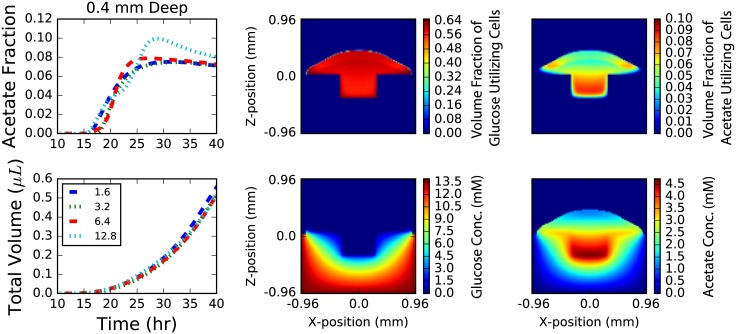
Hole geometry. Results for colonies grown at the bottom of a square hole in an agar surface 0.4 mm deep. Results for holes 0.2 mm deep are not shown as they are similar. The colony growth (top left) is mildly dependent upon the width of the hole with wider holes growing slightly more quickly. The relative fraction of acetate (bottom left) utilizers in the population grow with time until the wall the colony reaches the wall and a burst of glucose utilizers are grown until the colony on the wall is thick enough to begin producing acetate utilizers once again. The confinement leads to larger fraction of acetate utilizers than seen on flat surfaces. Snapshots of population volume fractions (top middle and right) and chemical concentrations (top middle and right) after 40 hours of growth. Further, a large build-up in acetate concentration (bottom middle; units of mM) inside the hole is apparent while nothing interesting occurs with the glucose concentration (bottom right; units of mM).

The behavior of colonies that grow in holes, on the other hand, are predicted to depend weakly on hole depth; however the aspect ratio plays a more interesting role. For narrow enough holes (low aspect ratio) the colony interacts with the glucose rich walls before acetate utilizing fractions begin to arise. High aspect ratio holes cause the growth of the acetate utilizing fraction to pause slightly until the colony has crested the edge and the distance from the agar (*e.g*. colony height) again drives growth in the acetate utilizing fraction (see Fig 9). The steady-state acetate utilizing fraction is generally larger than for flat colonies, laying somewhere around 8%. The hole geometry is particularly representative of a wound geometry; nutrients flow from the substrate and the colony top is exposed to oxygen. It has recently been shown in 1D simulations of diseased wound biofilm composed of *Pseudomonas aeruginosa* and *Staphylococcus aureus* that spatial partitioning (and colony stability) is dependent on cross-feeding and nutrient availability [20, 21]. It would be interesting to investigate how a 3D structure more representative of real wounds would change the results of these studies.

In general, holes and plateaus are both predicted to drive larger fractions of the colony into acetate consumption, but these effects are due to different reasons. In holes, a significant fraction of the colony grows very far from any oxygen, allowing acetate to build up inside the colony driving a larger fraction into acetate utilization state. This is quite apparent from examining images of the simulations (see Fig 6). When grown on plateaus, the limitation of glucose causes the large acetate utilizing fraction of cells near the column. It is easy to imagine how more complex arrangements of plateaus and holes will exhibit even more complex dynamics. Complex arrangements are easy to implement in microfluidic devices; therefore, testing the results of these simulations—and therefore the validity of the 3DdFBA model—should be relatively straightforward.

#### Concave and convex

Natural surfaces tend to curve. Curvature is especially important in human microbiome research as essentially all internal (*i.e*. gut, mouth, stomach, *etc*.) and external (*i.e*. skin, eye, nose, eardrum *etc*.) surfaces exhibit curvature on the length scales of millimeters to centimeters. We simulated *E. coli* growing on concave and convex agar surfaces with varying curvature. Concave surfaces are particularly interesting as they are quite analogous to a surface wound; nutrients flow up from inside the body while oxygen flows from above. While *E. coli* is not a major chronic wound pathogen, understanding the effects of curvature are nevertheless interesting. Simulations were run with curvatures ranging from about 0.5 (2mm radius) to about 1.0 (1 mm radius) in a single dimension (cylinder-like geometry).

Colony growth rate was approximately linearly related to curvature over the 30 hours of simulated time (see S5 and S6 Figs). While higher curvatures introduced relatively small increases in growth on concave surfaces (<2% compared to flat surfaces after 30 hours), they impeded growth on convex surfaces (up to 5% in the same amount of time). Our results demonstrate that a curved surface has relatively insignificant effect on the colony growth when compared to walls, plateaus and holes. This suggests that simulations of biological systems need not necessarily capture the exact geometry of a system, but rather the major features arising abrupt changes to colony confinement.

## Conclusion

Here we have performed the first parametric study of the effects of strain specific features and substrate geometry on the growth of *E. coli*. Using 3DdFBA, a multi-scale method coupling reaction-diffusion with FBA, we were able to elucidate subtle differences in the growth and metabolic cross-feeding of colonies growing on various agar surfaces due to varying initial conditions. Three observations are particularly important for future studies.

First, by examining the dependence of the solution on grid resolution we determined that a lattice spacing of ∼10 μm (or smaller) is required to ensure a converged solution. While larger resolutions get the overall colony growth more or less correct, larger errors in the extent of cross-feeding arise. And in fact, with grid resolutions of greater than about 30 μm, oscillatory patterns arise in the solution, suggesting numerical instability. While we acknowledge that the actual solution depends on the relative rates of diffusion, reaction and regulation, we nevertheless suggest future studies use a grid spacing of ∼10 μm when making quantitative predictions about metabolic cross-feeding.

Second, metabolic cross-feeding can depend on strain specific characteristics, even for nearly (genetically) identical organisms. As we found for five *E. coli* strains, the onset time of metabolic cross-feeding and the partitioning of the colony between metabolic phenotypes depend non-linearly on features such as growth, uptake and efflux rates for shared metabolites. These subtle differences could give rise to drastically different behavior when growing in consortia of competing organisms. Hypothetically, if another organism were to compete for acetate, *E. coli* strains W3110 and BL21 might not fractionate into different phenotypes, while the Crooks and W strains might. This behavior will likely be highly dependent on the actual scenario simulated; as a recent study of microbiome associations showed, the extent of cooperation versus competition is highly dependent on the concentration of available nutrients [33]. This conjecture could be easily verified via further 3DdFBA simulations.

Third, by examining various idealised geometries we were able to identify which features significantly impacted cross-feeding. Colonies growing on “hard” geometries near walls, edges, holes and plateaus resulted in significant differences compared to growth on flat surfaces. Partitioning of the population between phenotypes could be significantly affected by geometry (*e.g*. plateau and hole). Additionally, such geometries could introduce transient deviations from “steady-state” growth which could last for more than 20 hours (depending on the particular geometry). These results are in contrast to those seen for “soft” geometries (convex or concave surfaces), which showed minor deviations of maximally 5% in growth. Overall, our results suggest that when simulating microbial communities, it may suffice to only capture abrupt geometric features (*i.e*. walls, turns, confinement, *etc*.) while neglecting minor features (*i.e*. rough surfaces, mild curvature, *etc*.).

We believe that even this relatively simple study demonstrates the utility of 3DdFBA; such multi-scale methods supplement experimental techniques that are limited in temporal and/or spatial resolution. The simplicity and speed of 3DdFBA (nearly real-time on a modern, inexpensive GPU [16]) means it could become a computational instrument in experimental and theoretical laboratories alike. That being said, there are a number of algorithmic improvements that need to be implemented; such as multi-GPU spatial decompositions (for example [34]), and the addition of an advection equation to the algorithm, such as has been proposed by Chen *et al*. [20].

This work paves the way for future work examining more complex systems like the human gut microbiome. Recent work already demonstrated the potential for cross-feeding in the gut microbiome in idealized populations [35]. Further, a number of tools/databases that identify metabolic cross-feeding from metagenomic datasets (such as MMinte [33] and AGORA [36]) are now available. These will help inform construction of realistic model gut communities. Such studies could lead to insights into community dynamics, and potentially, their connection to disease.

## Supporting information

S1 Fig Code performance. Benchmarks for the 3DdFBA code used in this manuscript on 3 NVIDIA GPU models: 1) GeForce GTX Titan X, 2) Tesla K80 and 3) GeForce GTX 780. (left) Hours of real time required to simulate an hour of colony growth as a function of the simulation volume. Simulations were performed with a 1 *μm* lattice spacing. The inset shows the performance of the three different GPUs in units of time (ns) per lattice sites per timestep. (right) Memory required for a simulation consisting of 2 cell types and 5 chemical species. Horizontal lines show memory limitations of the GPUs indicated in the inset. Current GPUs can support simulations of over 100 mm^3^.
(EPS)

S2 Fig Concentration and fluxes for different *E. coli* strains. Colony cross-sections for the five strains depicting concentrations (top three rows) and fluxes (bottom three rows) for key metabolites after 30 hours of growth. Concentrations are reported in units of mM while fluxes in units of mmol/gDCW/hr.
(EPS)

S3 Fig Colony expansion for different *E. coli* strains. Growth of *E. coli* colony radius (left) and height (middle) initially show an exponential character limited by cell mass, prior to transitioning to a linear regime where nutrient availability is the primary factor limiting growth. Variability in nutrient uptake rates among the strains causes differences in the colony aspect ratios early in colony growth (right).
(EPS)

S4 Fig Relative acetate turnover. Colony volume normalized acetate consumption flux (*e.g*. *mmol*/*L*/*hr*) relative to strain MG1655.
(EPS)

S5 Fig Concave surface. Behavior of *E. coli* colonies grown on a concave surface containing metabolizable glucose. Different curves indicate different curvatures of the substrate. (top left) Colonies grown on substrate with higher curvature grew slightly faster after many hours of growth. (top left) The fraction of acetate utilizers in a community (relative to a colony growing on a nearly flat surface) depends linearly, but inconsequentially on the substrate curvature. A profile the colony showing the acetate utilizing fraction (contour level = 0.05; bottom right) and the whole colony (contour level = 0.64; top right) after 35 hours of growth. Colony volume and acetate fraction are shown relative to a colony grown on a surface with a curvature *κ* = 1.92.
(EPS)

S6 Fig Convex surface. Behavior of *E. coli* colonies grown on a convex surface containing metabolizable glucose. Different curves indicate different curvatures of the substrate. (top left) Colonies grown on substrate with higher curvature grew slightly slower after many hours of growth. (top left) The fraction of acetate utilizers in a community (relative to a colony growing on nearly flat surface) depends linearly, but inconsequentially on the substrate curvature. A profile the colony showing the acetate utilizing fraction (contour level = 0.05; bottom right) and the whole colony (contour level = 0.64; top right) after 35 hours of growth. Colony volume and acetate fraction are shown relative to a colony grown on a surface with a curvature *κ* = 1.92.
(EPS)

S1 Table Correlations. Correlations of maximal acetate utilizing fraction (left two columns) and onset time of acetate utilization (right two columns) with various bulk strain characteristics. Values that are significant with a (two-tailed) P-value ≤ 0.01 are indicated in bold.
(TIFF)
