# Correction: Parametric studies of metabolic cooperativity in *Escherichia coli* colonies: Strain and geometric confinement effects

**DOI:** 10.1371/journal.pone.0190193

**Published:** 2017-12-19

**Authors:** Joseph R. Peterson, John A. Cole, Zaida Luthey-Schulten

The following information is missing from the Funding section: J.R.P. and J.A.C. received travel funding under National Science Foundation Grant (PoLS) PHY-1026550.
