# Supplementary figures and images for: Parametric studies of metabolic cooperativity in Escherichia coli colonies: Strain and geometric confinement effects
Source: PLoS One. 2017 Aug 18;12(8):e0182570. doi: 10.1371/journal.pone.0182570 (PMC5562313; doi:10.1371/journal.pone.0182570)

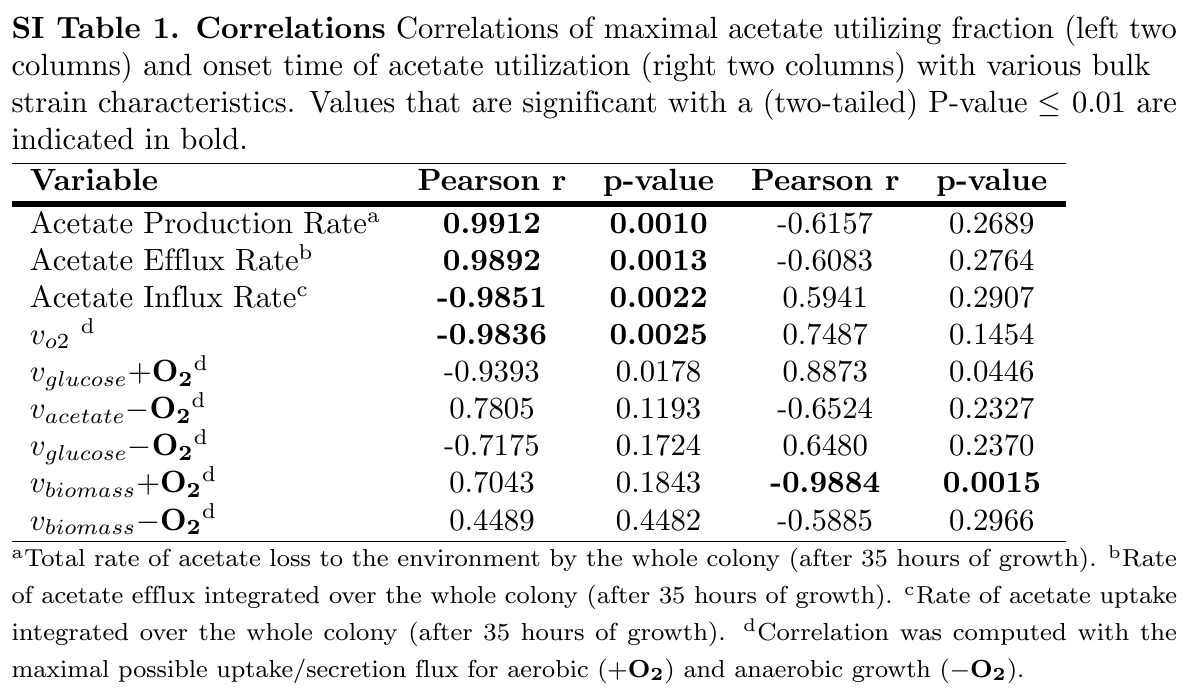

Supplement: S1 Table — Correlations of maximal acetate utilizing fraction (left two columns) and onset time of acetate utilization (right two columns) with various bulk strain characteristics. Values that are significant with a (two-tailed) P-value ≤ 0.01 are indicated in bold. (TIFF) [file pone.0182570.s007.tiff]
